# Species-dependent activities of the PINK1–parkin axis

**DOI:** 10.1186/s40035-026-00586-w

**Published:** 2026-09-18

**Authors:** Wei Huang, Kecheng Chen, Jiayi Wen, Tong Zhang, Shihua Li, Xiao-Jiang Li, Weili Yang

**Affiliations:** 1 State Key Laboratory of Bioactive Molecules and Druggability Assessment, Guangdong Key Laboratory of Non-human Primate Research, Key Laboratory of CNS Regeneration (Ministry of Education), GHM Institute of CNS Regeneration, Jinan University, Guangzhou, 510632 China; 2 Lingang Laboratory, Shanghai, 200031 China

**Keywords:** PINK1, parkin, Parkinson’s disease, Animal models, Non-human primate, Species specificity

## Abstract

In vitro studies have established that PTEN-induced putative kinase 1 (PINK1) and parkin are central regulators of mitophagy, and loss-of-function mutations in either gene can cause early-onset Parkinson’s disease (PD). Although various animal models, including mice and pigs with *PINK1* or *PRKN* knockout, have largely failed to recapitulate the neurodegeneration seen in PD patients, effective knockdown of *PINK1* or *PRKN* in non-human primates, when achieving substantial protein depletion, does induce dopaminergic neuron loss in the substantia nigra and α‑synuclein pathology, suggesting that both the degree of protein loss and the species-dependent PINK1–parkin axis activity contribute to PD pathogenesis. This review compares pathological and behavioral outcomes of PINK1- and parkin-deficient animal models across species, illustrates diverse functions of the PINK1–parkin axis beyond mitophagy, discusses mechanisms underlying the species-dependent differences, and highlights the therapeutic potential of targeting this pathway. The review also underscores the necessity of considering species-specific mechanisms when investigating the PINK1/parkin pathway.

## Introduction

Parkinson’s disease (PD) is a progressive neurodegenerative disorder characterized by a loss of dopaminergic neurons in the substantia nigra pars compacta and formation of Lewy bodies. Patients exhibit motor symptoms (bradykinesia, rigidity, tremor) and non-motor symptoms (hyposmia, REM sleep behavior disorder, depression, constipation) [1, 2]. The incidence of PD has increased over the past two decades, with the highest prevalence in Europe (1.80 per 1000) [3]. Pathogenesis involves mitochondrial dyshomeostasis, lysosomal dysfunction, and neuroinflammation [4]. In addition to sporadic PD, hereditary forms exist, with multiple associated genes identified (Table 1). Among these, PTEN-induced putative kinase 1 (*PINK1*) and *PRKN* are definitive causative genes for autosomal-recessive early-onset PD (EOPD) and serve as critical entry points for investigating PD pathogenesis.

**Table 1:** Genetic contributors to Parkinson’s disease (PD)

Gene	Classification	Genetic locus	Type of PD	Function	References
*SNCA*	Established PD gene	4q21.3-q22	AD/EOPD	Synaptic vesicle regulation	[5, 6]
*PRKN*	Established PD gene	6q25.2-q27	AR/EOPD	Mitochondrial autophagy	[7]
*UCH-L1*	Candidate gene	4p13	AD	Ubiquitin recycling	[8]
*PINK1*	Established PD gene	1p36.12	AR/EOPD	Mitochondrial autophagy	[9, 10]
*DJ-1*	Established PD gene	1p36.23	AR/EOPD	Oxidative stress protection	[11]
*LRRK2*	Established PD gene	12q12	AD/LOPD	Kinase regulating vesicle trafficking	[12, 13]
*ATP13A2*	Complex Parkinsonian phenotypes	1p36	AR/EOPD	Lysosomal cation transport	[14]
*PLA2G6*	Complex Parkinsonian phenotypes	22q13.1	AR	Lipid metabolism	[15]
*FBXO7*	Complex Parkinsonian phenotypes	22q12.3	AR	Ubiquitin–proteasome pathway	[16]
*VPS35*	Established PD gene	16q12	AD/LOPD	Retromer-mediated protein sorting	[17, 18]
*DNAJC6*	Complex Parkinsonian phenotypes	1p31.3	AR/EOPD	Clathrin-mediated endocytosis	[19]
*SYNJ1*	Complex Parkinsonian phenotypes	21q22.11	AR	Synaptic vesicle recycling	[20]
*DNAJC13*	Complex Parkinsonian phenotypes	3q22.1	AD	Endosomal trafficking	[21]
*CHCHD2*	Established PD gene	7p11.2	AD/LOPD	Mitochondrial respiratory regulation	[22]
*VPS13C*	Complex Parkinsonian phenotypes	15q22.2	AR	Lipid transport at organelle contacts	[23]
*PSAP*	Candidate gene	10q22.1	AD	Lysosomal sphingolipid degradation	[24]
*RAB32*	Complex Parkinsonian phenotypes	6q24.3	AD/LOPD	Intracellular membrane trafficking	[25, 26]
*GBA*	Complex Parkinsonian phenotypes	lq22	AR/LOPD	Lysosomal glucocerebroside degradation	[27]
*MAPT*	Candidate gene	17q21.31	AD	Microtubule stabilization	[28]
*NR4A2*	Candidate gene	2q24.1	AD/EOPD	Transcriptional regulator	[29]
*RAB39B*	Complex Parkinsonian phenotypes	Xq28	X-linked recessive	Intracellular membrane trafficking	[30]
*DCTN1*	Complex Parkinsonian phenotypes	2p13.1	AD	Microtubule-based intracellular transport	[31]
*GCH1*	Complex Parkinsonian phenotypes	14q22.2	AD/EOPD	Tetrahydrobiopterin (BH4) biosynthesis	[32]
*ATXN2*	Candidate gene	12q24.12	AD/LOPD	RNA metabolism regulation	[33]
*ITSN1*	Candidate gene	21q22.11	AD	Clathrin-mediated endocytosis	[34]

*AD* Autosomal dominant, *AR* Autosomal recessive, *EOPD* Early-onset Parkinson’s disease, *LOPD* Late-onset Parkinson’s disease

The PARK6 locus was initially mapped in families with early-onset parkinsonism, and subsequent studies identified *PINK1* as the causative gene underlying PARK6-linked parkinsonism [9, 35]. PINK1 is a 581-amino-acid protein kinase with protective cellular functions [35, 36], accounting for 1%–7% of EOPD cases [37]. In 1997, mutations in *PRKN*, which encodes parkin, were identified as the cause of autosomal recessive juvenile Parkinsonism in Japanese families [38]. Subsequently, *PRKN* mutations were found to be the most common cause of EOPD, accounting for 10%–20% of cases [39]. Parkin is a 465-amino-acid E3 ubiquitin ligase comprising an N-terminal ubiquitin-like (Ubl) domain and a C-terminal ubiquitin ligase domain [7, 40]. Patients with *PINK1* or *PRKN* mutations exhibit the typical PD pathology, dopaminergic neuron death in the substantia nigra (SN), and some postmortem brain investigations have also revealed the presence of α-synuclein (α-syn) aggregation [10, 41–43].

**Fig. 1:**
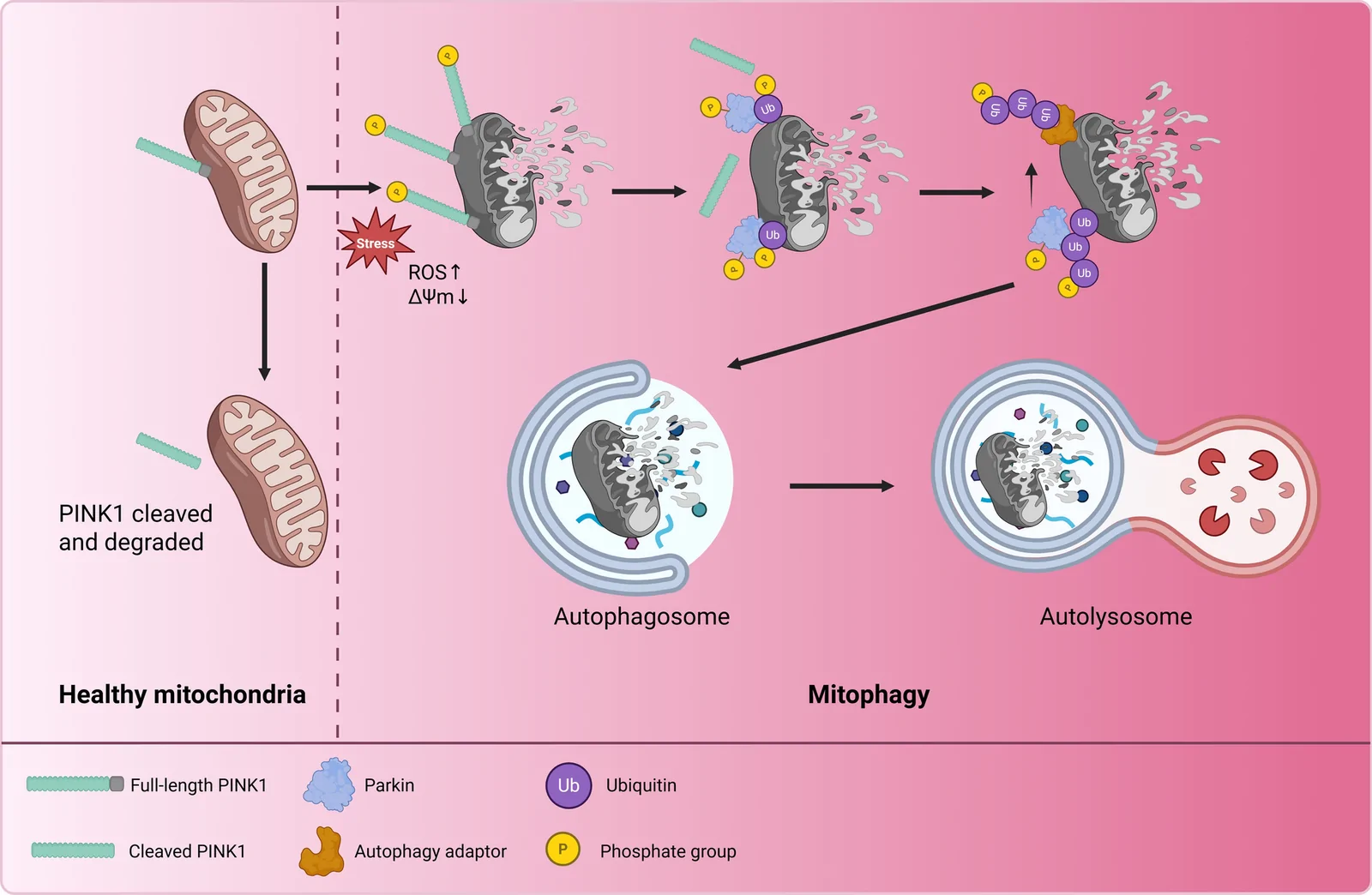
PINK1–parkin-mediated ubiquitin-dependent mitophagy. In healthy mitochondria, PINK1 is rapidly cleaved and degraded. Mitochondrial damage, marked by loss of membrane potential (ΔΨm) and reactive oxygen species (ROS) accumulation, stabilizes PINK1 on the outer mitochondrial membrane. The stabilized PINK1 then phosphorylates both ubiquitin and parkin, activating parkin and recruiting it to the damaged mitochondrion. Once there, parkin ubiquitinates outer membrane proteins, forming ubiquitin chains recognized by autophagy adaptors. These adaptors encapsulate the damaged mitochondrion into an autophagosome, which fuses with a lysosome for degradation. This process is essential for maintaining mitochondrial integrity and cellular homeostasis

Mitophagy is the primary pathway for eliminating dysfunctional mitochondria. In vitro studies have demonstrated that PINK1 and parkin are closely linked to ubiquitin-dependent mitophagy (Fig. 1). In cultured cells under normal conditions, PINK1 is imported into mitochondria and cleaved by PARL (Presenilin-Associated Rhomboid-Like protein), followed by proteasomal degradation [44]. Upon mitochondrial damage, PINK1 accumulates on the outer mitochondrial membrane, becomes activated, and phosphorylates ubiquitin [45] and parkin at Ser65 [46], recruiting and activating parkin [47–49]. Parkin then translocates to mitochondria [50–52] and ubiquitinates outer membrane proteins, triggering autophagosome formation and lysosomal degradation of damaged mitochondria and other proteins [53, 54]. Beyond orchestrating bulk mitophagy, the PINK1–parkin axis regulates mitochondrial quality control through the formation of mitochondrial-derived vesicles (MDVs). As a localized surveillance mechanism, MDVs selectively sequester and transport damaged proteins or lipids for degradation without necessitating the elimination of the entire organelle [55]. This process operates independently of the canonical autophagy machinery, highlighting the multi-faceted nature of PINK1–parkin-mediated mitochondrial maintenance. Recent evidence further clarifies the molecular coordination of these pathways, revealing how cells dynamically balance mitophagy and MDV biogenesis in response to varying degrees of mitochondrial stress [56]. In addition to regulating mitophagy and MDV trafficking, the PINK1–parkin pathway has been implicated in mitochondrial antigen presentation, antimicrobial defense, and gut–brain axis-associated neuroinflammation [57–59]. These findings broaden current pathogenic models of PD beyond mitochondrial dysfunction and α-syn pathology, although their contribution to human disease remains incompletely understood.

Because direct human tissue studies are ethically constrained, animal models remain indispensable for investigating how *PINK1* and *PRKN* mutations contribute to PD pathogenesis. However, various animal models expressing *PINK1* or *PRKN* mutations display divergent phenotypes, and species-specific differences must be carefully considered [60]. This review covers four main scopes: (1) summarizing studies in non-mammalian models, mice, and non-human primates to investigate the PINK1/parkin axis; (2) comparing findings across these models; (3) emphasizing the value of non-human primate models; and (4) discussing challenges for studying pathogenic mechanisms and developing therapies.

## The PINK1–parkin pathway in *Drosophila* models and other non-mammalian models

The PINK1/parkin axis was firstly identified in *Drosophila*. Fruit flies offer advantages: ease of culture, well-characterized behaviors, simple genetics, and orthologs for many human disease genes [61]. Approximately 75% of human disease genes have fly counterparts [62, 63], including PD-related genes *PRKN* [64], *PINK1* [65], *LRRK2* [66], and *SNCA* [67]. Flies possess an organized dopaminergic neuron system, enabling PD modeling [68].

The *Drosophila* parkin ortholog shares 42% identity and 59% similarity with human parkin. *parkin* transcripts are detected throughout development, with highest levels in adults [69]. *parkin* mutations cause reduced lifespan, male sterility, abnormal mitochondrial morphology, and locomotor deficits, though tyrosine hydroxylase (TH)-positive cell loss is restricted to the dorsomedial dopaminergic cluster. *Drosophila* *Pink1* encodes a 721-amino acid polypeptide with a mitochondrial targeting motif and a serine/threonine kinase domain, showing 42% similarity (1% identity) to human PINK1 [35, 36]. *Pink1* loss-of-function mutants (*Pink1*^*D3*^, *Pink1*^*B9*^, *Pink1*^*RV*^) exhibit phenotypes similar to *parkin* mutants: reduced lifespan, mitochondrial abnormalities, male sterility, and locomotor deficits. Double *Pink1* and *parkin* mutants show the same muscle phenotypes as single mutants, confirming that Pink1 and parkin act in the same pathway. Additionally, *Pink1* mutations are rescued by *parkin* overexpression, suggesting that Pink1 acts upstream [36, 70, 71]. The axis also influences mitochondrial DNA localization [72], endoplasmic reticulum (ER) stress [73], and calcium uptake [74]. Recent evidence suggests involvement of innate immune signaling, as STING deletion can rescue mitochondrial and muscle defects in parkin mutant *Drosophila* [75].

Importantly, the phenotypic consequences of PINK1/parkin dysfunction are markedly more severe in *Drosophila* [36, 70] compared with mammalian models [76], in which baseline dopaminergic neuron loss and mitochondrial abnormalities are often more modest or context-dependent, likely reflecting stronger compensatory capacity in higher organisms.

Beyond *Drosophila*, other non-mammalian models have also contributed to understanding PINK1/parkin biology. In *Caenorhabditis elegans*, genetic studies have revealed conserved roles of the pathway in mitochondrial quality control and stress responses, while recent work has further highlighted its utility for therapeutic screening [77]. In zebrafish, Pink deficiency disrupts dopaminergic neuronal organization and motor behavior despite limited neuronal loss [78]. These models have proven valuable for investigating mitochondrial dysfunction and evaluating candidate therapeutics in vivo.

Collectively, these non-mammalian systems have been instrumental in elucidating conserved PINK1/parkin biology and identifying potential therapeutic strategies. However, their translational relevance is constrained by genetic, neuroanatomical, and pathological differences from humans. Most notably, they lack the selective vulnerability of SN dopaminergic neurons and do not faithfully recapitulate hallmark PD pathologies such as Lewy body formation. Even in *Drosophila*, some studies report no loss of DA neurons or TH reduction in *parkin* knockout (KO) flies [69, 70, 79], and the basal mitophagy is unaffected by *Pink1* or *parkin* knockout [80].

## Lack of robust phenotypes of PINK1/Parkin knockout rodent models

Mice are widely used due to the ease of genetic manipulation, short generation time, low cost, and evolutionary proximity to humans. Numerous *Pink1* and *Prkn* KO mouse models have been generated (Tables 2 and 3).

**Table 2:** Phenotypes of *Prkn*-knockout mice

Model	Phenotypes	References
*Prkn* exon3 KO	In mouse models with knockout of *Prkn* or *Pink1*, there are no significant differences in TH levels in the SN and striatum, indicating no apparent loss of dopaminergic neurons in the nigrostriatal pathway. However, there is a decrease in dopamine levels in the striatum	[81]
*Prkn* KO	No significant morphological abnormalities in the mitochondria, but a reduction in mitochondrial respiration	[82]
*Prkn* exon7 KO	The nigrostriatal dopaminergic system does not show any damage, and there are no significant differences in motor phenotypes	[83]
*Prkn* exon2 KO	The dopaminergic neurons in the substantia nigra pars compacta do not show signs of degeneration, and there are no differences in behavioral aspects	[84]
*Prkn* exon3 KO	Only mild behavioral/motor deficits, with increased extracellular dopamine, but without loss of dopaminergic neurons in the SN	[85]
*Prkn* exon3 KO	No obvious motor phenotypes, abnormalities in DA metabolism, or signs of degeneration in the nigrostriatal pathway	[86]
*Prkn* exon2 KO	No significant changes in motor behavior; the number and morphology of dopaminergic neurons are normal; levels of dopamine, dihydroxyphenylacetic acid, and homovanillic acid remain unchanged	[87]
*Prkn* exon2 KO	No obvious dopaminergic neurodegeneration in young mice. At 110 weeks of age, mice develop motor deficits, including hindlimb abnormalities, accompanied by loss of dopaminergic neurons, mitochondrial fragmentation, abnormal mitochondrial ultrastructure, and mitochondrial accumulation in surviving neurons, indicating age-dependent neurodegeneration associated with impaired mitochondrial quality control	[88]
*Prkn* exon2 KO	Normal lifespan and fertility; no overt motor deficits, dopaminergic neurodegeneration, mitochondrial respiratory defects, or mtDNA depletion during aging; normal OXPHOS function in the brain, heart, and skeletal muscle	[89]
Adult DA neuron-specific *Prkn* KO	No motor impairment, nigral neurodegeneration, or major transcriptional alterations in dopaminergic neurons following adult-onset parkin deletion	[89]
Adult conditional *Prkn* KO	Progressive DA neuron degeneration driven by PARIS accumulation and suppression of PGC-1α-dependent mitochondrial biogenesis; neurodegeneration is PARIS-dependent and rescued by PGC-1α restoration	[90]

**Table 3:** Phenotypes of *Pink1* knockout mice

Model	Phenotypes	References
*Pink1* KO	Mitochondria in the striatum do not exhibit any significant structural defects, and the levels of oxidative stress markers remain unchanged	[91]
Targeting intron V	No visible aggregates of α-syn or neurodegeneration in the SN and striatum. Normal mitochondrial quality and structural integrity are maintained	[76]
*Pink1* exon4-7 KO	Dopaminergic neuronal number and levels of dopamine and dopamine receptors in the striatum remain unchanged	[92]
*Pink1* exon4-5 KO	A significant decrease in striatal dopamine in the absence of any significant loss of dopaminergic neurons at 6 months of age	[93]
*Pink1* exon1 KO	Mitochondrial ATP levels in the striatum are decreased	[94]
*Pink1* exon2-3 KO	No significant changes in motor behavior, with some alterations in mitochondrial morphology	[95]
*Pink1* KO	No significant changes in TH-positive neurons in the substantia nigra pars compacta	[96]
*Pink1 KO*	No overt nigral dopaminergic neuron loss or severe motor deficits under basal conditions	[97]
*TH-dOTC/Pink1* KO	Exacerbated dopaminergic neurodegeneration and motor dysfunction compared with TH-dOTC mice, accompanied by loss of L-DOPA responsiveness, indicating that PINK1 protects against mitochondrial stress-induced neurodegeneration	[97]
*Pink1* KO	Minimal spontaneous neurodegeneration and Lewy pathology under basal conditions	[98]
*APP/PS1::Pink1−/−*	Spontaneous Lewy body-like pathology at endogenous α-syn levels, widespread α-syn aggregation in central and peripheral nervous systems, behavioral abnormalities, and heterogeneous DLB-like phenotypes	[98]
Adult conditional *Pink1* knockdown	Progressive degeneration of nigral dopaminergic neurons, reduced PGC-1α expression, PARIS accumulation, and astrogliosis. Neuronal loss is rescued by human PINK1 overexpression or PARIS knockdown, indicating a PARIS-dependent mechanism	[99]

However, no severe mitochondrial defects or dopamine abnormalities are observed in *Prkn* KO mice [100–102]. Similarly, *Pink1* KO mice show only mild mitochondrial defects and no pronounced neuronal death [76]. Even triple knockouts (*Pink1*, *Prkn*, *Park7*) lack significant neurodegeneration [103]. Most studies of *Pink1* or *Prkn* KO mice failed to recapitulate the massive dopaminergic neurodegeneration or motor deficits seen in PD patients. The short lifespan of mice may also limit modeling of human neurodegenerative diseases [100].

These observations further suggest that the relatively mild phenotypes observed in rodent models cannot be explained solely by defects in mitophagy. Additional PINK1/parkin-dependent functions may also contribute to neuronal vulnerability. Notably, endogenous PINK1 protein is unstable and difficult to detect in the mouse brain [104], which may explain why its low endogenous levels fail to replicate the mitophagy functions observed in cultured cells following overexpression in vitro [105]. Indeed, basal mitophagy persists in *Pink1*-deficient mice [106] and *parkin*- or *Pink1*-deficient flies [80, 107], indicating that parkin and PINK1 are not essential for physiological mitophagy in small animals. Moreover, recent studies in neurons suggest that there are multiple mitochondrial quality control pathways operating in parallel or independently of PINK1/parkin, and that their contribution to the basal mitochondrial turnover remains context-dependent [108]. Additionally, PINK1–parkin functions independent of mitophagy have also been reported [109, 110]. Importantly, although the PINK1/parkin pathway has historically been characterized primarily as a regulator of mitophagy, accumulating evidence now supports a broader role in mitochondrial quality control, encompassing not only mitochondrial clearance but also mitochondrial biogenesis, transcriptional regulation [99], and suppression of neuroinflammatory responses [111]. In this regard, growing evidence indicates that PINK1/parkin signaling can regulate mitochondrial biogenesis through substrates such as parkin interacting substrate (PARIS, ZNF746) and downstream effectors including peroxisome proliferator-activated receptor gamma coactivator-1-alpha (PGC-1α) [90, 99]. In rodent models, loss of PINK1 or parkin leads to PARIS accumulation, repression of PGC-1α-dependent transcriptional programs, impaired mitochondrial biogenesis, and progressive dopaminergic neurodegeneration, which can be rescued by restoring PGC-1α activity [90]. These findings suggest that defects in mitochondrial biogenesis, in addition to impaired mitophagy, may contribute to dopaminergic neuron vulnerability.

At the same time, evidence from rodent models suggests that the pathological consequences of PINK1/parkin dysfunction may depend on the presence of additional genetic, environmental, or age-related stressors. Isolated PINK1 or parkin deficiency generally results in relatively mild or slowly progressive phenotypes, suggesting the existence of compensatory mechanisms that maintain neuronal homeostasis. However, additional pathogenic stressors can unmask substantial neurodegeneration. For example, PINK1 deficiency increases the susceptibility to α-syn-induced mitochondrial dysfunction and neuronal injury [112], and can promote Lewy body-like pathology in the presence of amyloid-related proteostatic stress [98]. Likewise, mitochondrial stress markedly exacerbates neurodegeneration in *Pink1*-deficient mice [97], highlighting a synergistic interaction between genetic susceptibility and environmental insults. Aging represents another important second hit, as aged parkin-deficient mice develop dopaminergic neuronal loss and mitochondrial abnormalities despite limited pathology at younger ages [88]. Representative examples are summarized in Tables 2 and 3. Collectively, these findings suggest that PINK1/parkin dysfunction establishes a vulnerable cellular state that frequently requires additional pathological stressors to overcome compensatory mechanisms and drive overt neurodegeneration.

Accordingly, although rodent models may not fully recapitulate the extent of neurodegeneration observed in PD patients, they remain highly valuable for dissecting gene–gene interactions, gene–environment interactions, and mechanisms underlying context-dependent disease susceptibility. However, because most functional studies of the PINK1–parkin axis have relied on in vitro systems and small animal models, whether this pathway exerts distinct functions in larger mammals, particularly non-human primates, remains unclear. Collectively, these findings highlight the importance of investigating PINK1/parkin biology in species more closely related to humans.

## Recent findings from large animal models

In the past decade, several research teams have turned to large animal models to investigate the functions of PINK1 and parkin. Pigs share some similarities with humans and have been used to model neurodegenerative diseases like Huntington’s [113]. However, *PINK1* and *PRKN* double-knockout pigs generated via CRISPR/Cas9 and somatic cell nuclear transfer did not exhibit typical PD motor deficits even at 7 months of age [114]. Similarly, *PARK7*/*PINK1*/*PRKN* triple-knockout pigs remained healthy with no behavioral deficits at 10 months [115]. This lack of robust phenotypes highlights the gap between genetic disposition and phenotypic manifestation.

The absence of PD phenotypes in pigs led researchers to turn to non-human primates. The monkey brain is more structurally similar to the human brain than the pig brain, and genetic similarity between rhesus macaques and humans reaches 93% [116]. Both PINK1 and parkin proteins share 97% similarity between the two species, making monkeys highly suitable for modeling PD. Beyond this genetic similarity, recent studies have revealed unique characteristics of endogenous PINK1 expression in the primate brain that may be particularly relevant to PD pathogenesis. Biochemical analyses have identified a predominant 55-kDa truncated form of PINK1 (PINK1-55) with kinase activity in both human and monkey brains. Unlike the transient mitochondrial PINK1 described under stress conditions, PINK1-55 is constitutively expressed in the primate brain and is enriched in membrane-associated fractions, including mitochondria and ER-related compartments. Furthermore, its expression is particularly prominent in the SN and brainstem, regions that are highly vulnerable in PD. These observations suggest that endogenous PINK1 may participate in broader neuronal functions in primates beyond its canonical role in mitophagy. The development of non-human primate models therefore provides a unique opportunity to investigate the physiological and pathological functions of endogenous PINK1 in the brain [117].

Chen et al. used a truncated sgRNA/Cas9-D10A nickase system delivered via Sendai virus to target *PINK1* exon 2 in cynomolgus monkey zygotes. PINK1 deficiency did not affect iPSC differentiation, and no neuronal loss or mitochondrial abnormalities were observed [118]. However, it remained unknown whether PINK1 reduction in the monkey brain was sufficient to cause neurodegeneration.

Subsequent studies demonstrated that PINK1 deficiency can cause neurodegeneration. Hu’s team used adeno-associated virus serotype 9 (AAV9)-delivered CRISPR/Cas9 to edit *PINK1* and *PARK7* in the SN of rhesus macaques. Six weeks post-injection, monkeys exhibited classic PD symptoms (tremor, bradykinesia, gait instability), severe dopaminergic neuron loss, and α-syn pathology [119]. Our research team achieved *PINK1* knockout in monkeys at both embryonic and adult stages using CRISPR/Cas9 targeting exons 2 and 4 of *PINK1* [120, 121]. PINK1 deletion impaired neuronal survival during both development and adulthood. Interestingly, no changes in mitochondrial structure, dynamics, or related protein markers were observed in *PINK1* mutant monkey brains [119, 120]. Instead, global downregulation of protein phosphorylation was observed—a phenomenon not seen in *Pink1* KO mice [122]. The severe neuronal loss in *PINK1* mutant monkeys contrasts sharply with the absence of neurodegeneration in *Pink1* KO mice, suggesting species-dependent PINK1 function.

Research on parkin in large animal models has been scarcer. In 2024, Han et al. disrupted exons 2 and 3 of the monkey *PRKN* gene using CRISPR/Cas9 [123]. Similar to *PINK1* knockout, parkin deficiency led to SN neurodegeneration and α-syn aggregation. Although parkin knockdown did not alter mitochondrial proteins, it reduced ^18^F-DOPA levels in the striatum and caused age-dependent neurodegeneration [123]. Notably, *PINK1* knockdown induces more severe neurodegeneration than parkin deficiency, suggesting independent functions of PINK1 and parkin. Indeed, under physiological conditions, PINK1 is enriched in membrane-bound organelles while parkin is soluble in the cytoplasm in the monkey brain, also supporting their independent roles [121].

Although in vitro evidence shows that PINK1 phosphorylates parkin at Ser65 to regulate mitophagy [46, 49], in vivo evidence in animal brains has been lacking. The *PRKN* knockdown monkey study provided the first in vivo evidence of PINK1-mediated parkin S65 phosphorylation in primate brains [123]. Most strikingly, this phosphorylation defect is associated with α-syn pathology. Analysis of the limited postmortem brain tissue from PD patients with *PINK1* or *PRKN* mutations has yielded inconsistent findings regarding α-syn pathology. While some studies report an absence of Lewy bodies [124–127], others demonstrate their presence in patients with *PINK1* mutations [10, 41, 43, 128] or *PRKN* mutations [129–131]. Notably, the pathological heterogeneity observed in patients does not fully align with the prominent α-syn pathology reported in some non-human primate models. This discrepancy may reflect differences in disease duration, compensatory capacity, genetic background, and experimental design. In humans, hereditary PD develops over decades, allowing adaptive mechanisms to emerge, whereas CRISPR/Cas9-mediated gene editing induces a more rapid loss of PINK1 or parkin function. In addition, patient-specific factors, including coexisting pathologies and genetic modifiers, may influence Lewy body formation. Therefore, differences between human patients and monkey models likely reflect biological and methodological factors rather than a fundamental mechanistic contradiction. Nevertheless, the identification of α-syn pathology in PINK1- and parkin-deficient monkeys provides a valuable framework for investigating the relationship between PINK1/parkin dysfunction and α-syn pathology in PD.

## Potential mechanisms for species-dependent functions of the PINK1–parkin axis

As discussed above, knockout of *PINK1* or *PRKN* produces different phenotypes across species. In non-human primates, knockout leads to significant neuronal loss, whereas mouse neurons are more tolerant. Mouse and pig brains also lack evidence of PINK1-mediated parkin phosphorylation, which contrasts with the results from primate brains [123, 132].

An alternative, but not mutually exclusive, interpretation is that the dependence on the PINK1/parkin pathway may exist across species but operate at different pathogenic thresholds. In rodents, loss of PINK1 or parkin alone often results in relatively mild phenotypes and may remain partially compensated for extended periods. However, additional stressors such as α-syn accumulation, mitochondrial dysfunction, environmental insults, or aging can unmask substantial neurodegeneration in these models [88, 97, 111]. In contrast, primate neurons may possess a lower threshold for PINK1/parkin dysfunction because of their greater metabolic demands, longer lifespan, and more complex neuronal architecture. Under this framework, non-human primate models may reveal a broader spectrum of disease manifestations, whereas rodent models remain highly valuable for dissecting gene–gene interactions, environmental modifiers, and mechanisms of disease susceptibility.

Another critical factor contributing to the species-dependent differences in the phenotypic consequences of PINK1/parkin dysfunction is the differential capacity for developmental and cellular compensation. In rodent models, early embryonic disruption of the PINK1/parkin pathway can induce robust compensatory adaptations within alternative mitochondrial quality control networks, thereby buffering the impact of pathway loss and resulting in relatively mild baseline phenotypes. In contrast, studies targeting downstream effectors of PINK1/parkin signaling, such as the PARIS/ZNF746 and PGC-1α biogenesis pathway, indicate that genetic or pathological disruption in mature, adult neurons—rather than during early development—is far more likely to exhaust these compensatory reserves and trigger adult-onset neurodegeneration [90, 99]. Consistent with this view, restoring PGC-1α activity effectively rescues mitochondrial biogenesis deficits and attenuates dopaminergic neuronal loss in mature systems [90, 99]. Notably, however, in non-human primate models, early single-gene knockout of *PINK1* is sufficient to induce progressive, spontaneous neurodegeneration under basal conditions. Together, these findings support the concept that the interspecies differences in the susceptibility to PINK1/parkin dysfunction fundamentally reflect differences in evolutionary compensatory capacity, where primates possess a significantly lower threshold for metabolic and transcriptional failure.

Species-dependent expression may further shape these pathways. In mice, Pink1 is rapidly cleaved and degraded under basal conditions, making it difficult to detect. In primate brains, however, PINK1 is abundantly expressed in neurons and glia, and this expression is markedly reduced by gene knockdown. PINK1-mediated phosphorylation of parkin, BAD [133], and other substrates is observed in monkey brain but not pig brain, where endogenous PINK1 is also undetectable. This species-dependent expression appears to be regulated at the protein level, rather than the mRNA level, as *PINK1* mRNA levels are comparable across species. Furthermore, the stable form of PINK1 (PINK1-55) in primate brains has a smaller size than full-length PINK1, having lost its N-terminal mitochondrial targeting domain while retaining kinase activity. How this PINK1-55 form is stably expressed to execute its kinase function remains to be investigated.

**Fig. 2:**
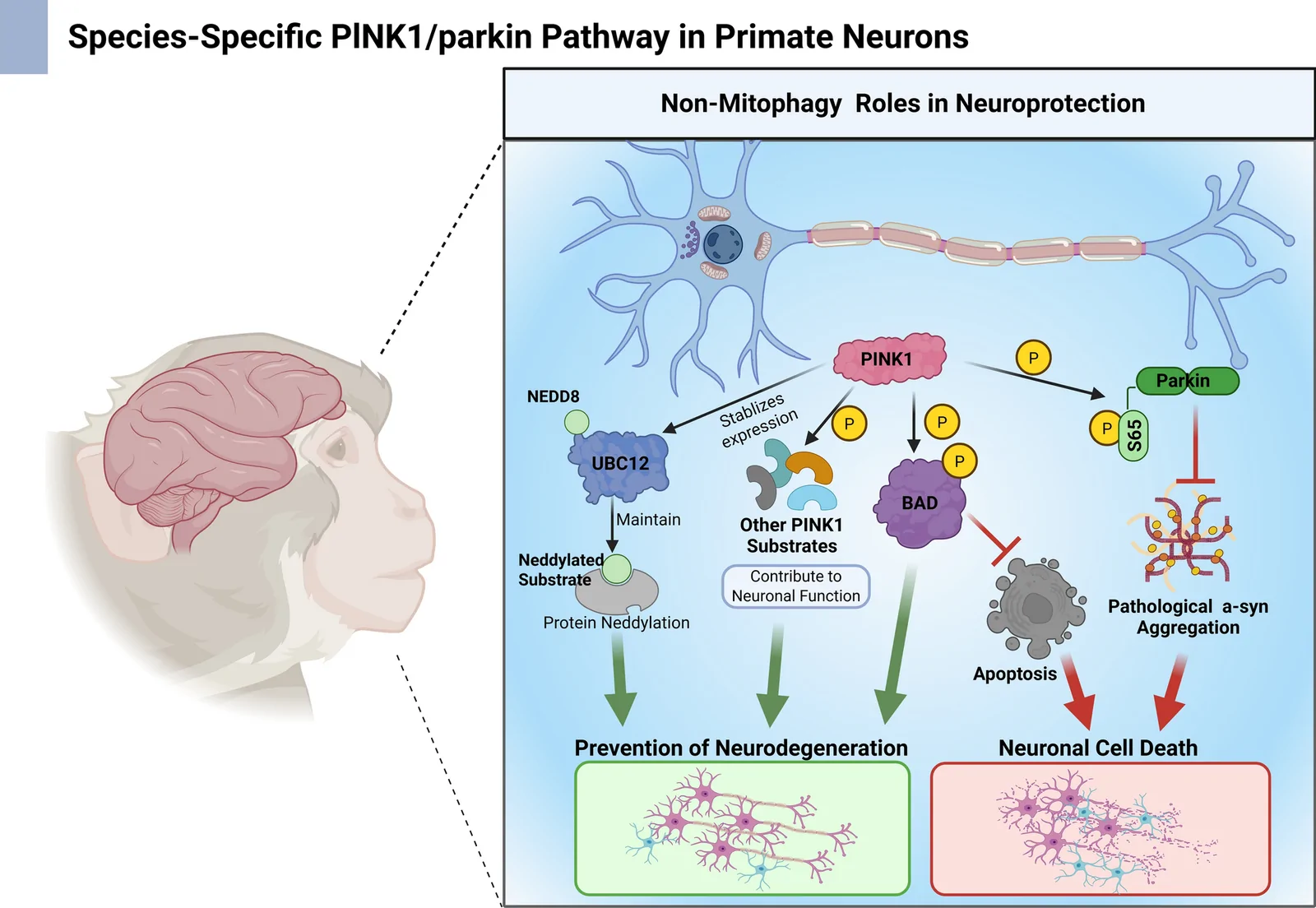
Species-specific roles of the PINK1/parkin pathway in primate neurons beyond mitophagy. In primate neurons, PINK1 may exert neuroprotective functions through multiple non-mitophagy mechanisms. In the primate brains, PINK1-mediated phosphorylation of parkin at Ser65 inhibits pathological α-synuclein aggregation. Additionally, PINK1 promotes protein neddylation by stabilizing UBC12 and phosphorylates BAD and other substrates critical for neuronal function, collectively preventing neurodegeneration. Disruption of these pathways compromises neuronal homeostasis and ultimately leads to cell death

In addition to the species-dependent expression, substrates and modulators of PINK1 and parkin may also differ across species. For example, PINK1 loss selectively reduces the expression and neddylation of ubiquitin conjugating enzyme 12 (UBC12) only in monkey brains [134]. Neddylation dysregulation is highly correlated with neurodegenerative diseases, and inhibiting neddylation induces cellular senescence and affects dopaminergic neuron survival [135, 136] (Fig. 2). Thus, non-human primates that express detectable endogenous PINK1 provide a new model to address the in vivo function of PINK1/parkin axis. However, direct evidence from human and non-human primate tissues remains limited, and most currently identified PINK1/parkin substrates have been characterized in human cellular models. Representative primate-relevant substrates and signaling pathways are summarized in Table 4.

**Table 4:** Potential PINK1/parkin substrates and signaling pathways in primate neurons

Substrate	Pathway/organelle	Primary function & signaling cascades	Evidence in primate samples	PINK1/parkin	References
parkin (Ser65)	Mitophagy/Mitochondria	Activates E3 ubiquitin ligase activity and initiates mitophagy	Reduced phosphorylation in mutant monkey brains; human recombinant parkin protein (in vitro)	Direct PINK1 substrate	[47]
Ubiquitin (Ser65)	Mitophagy/Mitochondria	Amplifies parkin-mediated ubiquitin signaling during mitophagy	Reduced phosphorylation in mutant monkey brains; demonstrated in human cell lines	Direct PINK1 substrate	[45]
BAD	Apoptosis/Mitochondria	Regulates BCL-2 family signaling and neuronal survival	Reduced phosphorylation in mutant monkey brains; demonstrated in human cell lines	PINK1 substrate	[133]
Drp1	Mitochondrial dynamics	Controls mitochondrial fission and quality control	Demonstrated in human cell lines	PINK1 substrate	[137]
UBC12	NEDDylation	Activates NEDD8 conjugation and Cullin-RING ligases	Identified as a PINK1-interacting protein in monkey brains	PINK1-associated interactor	[134]
STEP61	Synaptic signaling	Regulates ERK1/2-CREB signaling, glutamate receptor trafficking and synaptic plasticity	Elevated in human PD striatum and AR-JP samples; demonstrated in human cell lines	parkin substrate	[138]
Pael-R (GPR37)	Endoplasmic reticulum	Misfolded membrane protein; accumulation induces ER stress and unfolded protein response	Accumulation reported in AR-JP pathology; demonstrated in human cell lines	parkin substrate	[139]
αSp22 (O-glycosylated α-syn)	Synaptic/Lewy body pathway	Modified form of α-syn implicated in protein aggregation and Lewy body pathology; links ubiquitin–proteasome system to α-syn turnover	Identified directly in human brain; accumulates in AR-PD brains lacking parkin	parkin substrate	[140]
Miro1 (RHOT1)	Mitochondrial outer membrane	Mitochondrial trafficking and arrest of axonal transport during mitophagy	Validated in human fibroblasts, human neuronal cells, and patient-derived cellular models	PINK1 phosphorylates Miro1; parkin ubiquitinates and degrades Miro1	[141]

## Integrating PD genetic risk loci into the species-specific PINK1/parkin landscape

Accumulating evidence suggests that the PINK1/parkin axis does not function in isolation but instead occupies a central position within a broader genetic network implicated in PD. Multiple PD-associated risk genes, including *GBA* (glucocerebrosidase), *LRRK2* (Leucine-rich repeat kinase 2), *VPS35*, *PARK7*, *VPS13C* (vacuolar protein sorting 13C), *MAPT*, and RAB-family proteins, converge on cellular quality-control pathways that intersect with PINK1/parkin-mediated mitochondrial homeostasis.

For example, *GBA* mutations impair lysosomal function and compromise mitochondrial quality control by disrupting the efficient clearance of damaged mitochondria through the autophagy–lysosome pathway [142, 143]. Although GBA is not a canonical component of the PINK1/parkin signaling cascade, the GBA-mediated lysosomal dysfunction directly limits the effectiveness of the PINK1/parkin-dependent mitophagy, supporting the concept of a shared mitochondrial–lysosomal quality-control network. Similarly, LRRK2 and VPS35 are functionally linked to the PINK1/parkin pathway through Rab-dependent vesicular trafficking. Pathogenic *LRRK2* mutations impair the PINK1/parkin-mediated mitophagy [144], whereas the *VPS35* D620N mutation disrupts mitochondrial quality control through enhanced LRRK2 kinase activity and aberrant Rab10 phosphorylation [145]. In parallel, recent studies indicate that DJ-1 acts as an essential downstream component of the PINK1/parkin-dependent mitophagy by facilitating OPTN (optineurin) recruitment to damaged mitochondria [146]. Additional functional intersections have been reported for VPS13C, which regulates organelle contact sites and mitochondrial homeostasis [23, 147], *MAPT*, with pathological tau accumulation modulated by PINK1-dependent quality-control pathways [148–150], and RAB-family proteins, which participate in PINK1- and parkin-regulated membrane trafficking and autophagosome formation [25, 151, 152].

**Fig. 3:**
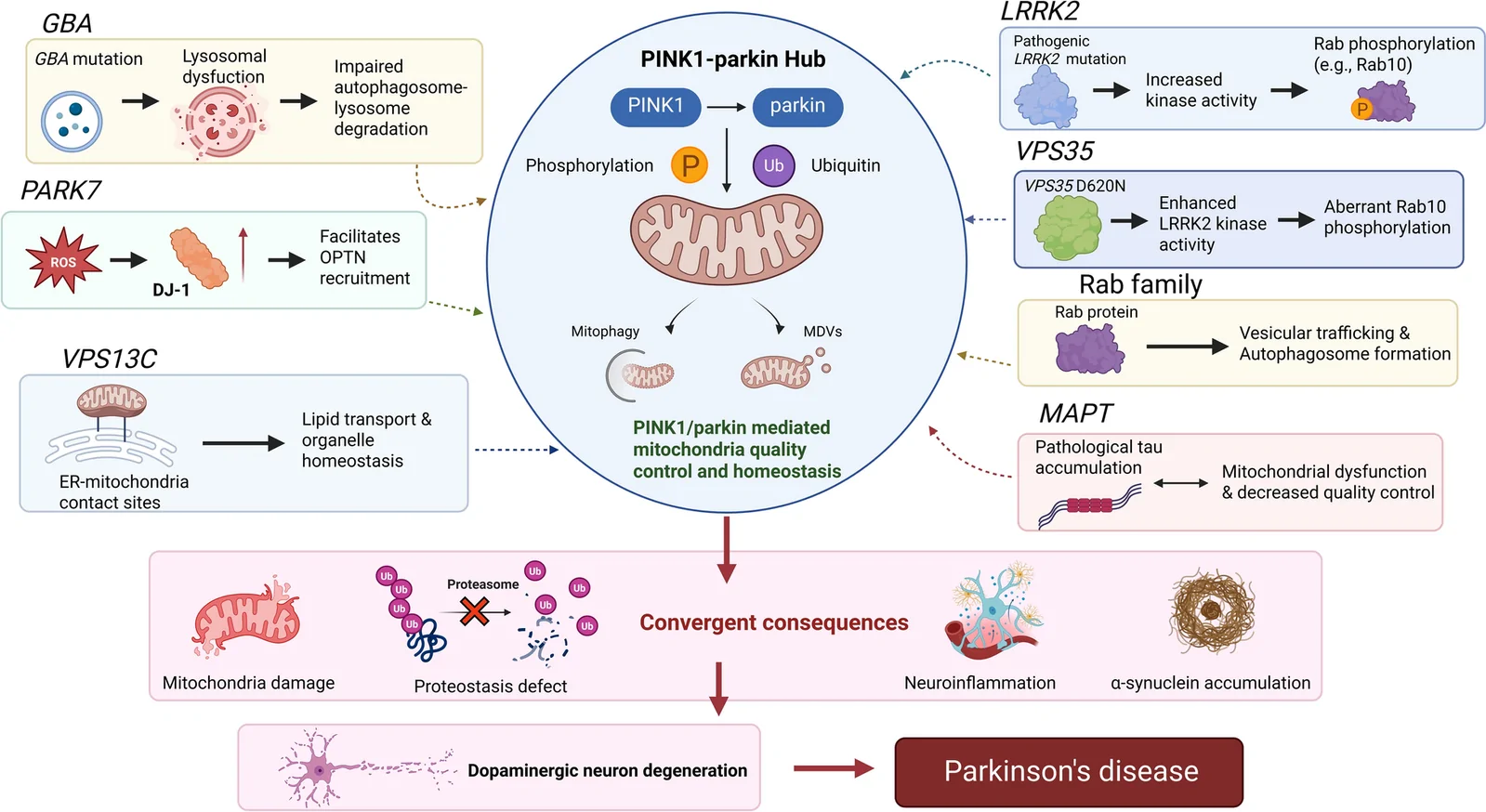
Integration of the PINK1/parkin quality-control hub with broader PD genetic networks and species-dependent pathogenic thresholds. At the cellular level, the PINK1/parkin axis acts as a central regulatory hub for mitochondrial homeostasis (mitophagy and MDVs). Diverse PD-associated risk factors converge on this pathway, including those affecting lysosomal clearance (*GBA*), vesicular trafficking (*LRRK2, VPS35*, RABs), organelle contacts (*VPS13C*), and oxidative stress responses (*DJ-1*). Failure of this integrated network triggers a convergent cascade of mitochondrial dysfunction, proteostasis failure, and α-synuclein aggregation, culminating in dopaminergic neurodegeneration

These observations indicate that PINK1/parkin serve as a convergent pathogenic hub integrating multiple PD risk pathways (Fig. 3). Importantly, interactions among these pathways may also contribute to the striking species-dependent phenotypes observed following PINK1 or parkin deficiency. In rodents, extensive compensatory mechanisms often buffer the consequences of isolated *Pink1* or *Prkn* deletion, resulting in relatively mild phenotypes. However, the introduction of additional genetic risk factors, aging, α-syn pathology, mitochondrial stress, or lysosomal dysfunction frequently unmasks substantial neurodegeneration. Such findings support the concept that the phenotypic threshold for PINK1/parkin dysfunction is determined not only by the pathway itself but also by the broader genetic and cellular context.

Conversely, primate neurons may operate closer to this pathological threshold because of their greater metabolic demands, extensive axonal arborization, and reliance on complex organelle quality-control mechanisms [153–156]. Recent studies have identified a primate-specific role of PINK1 in regulating UBC12 expression and protein neddylation, whereas comparable regulation of this pathway by Pink1 has not yet been reported in rodent models [134]. Therefore, species differences in PINK1/parkin-related phenotypes likely reflect both intrinsic differences in PINK1/parkin biology and distinct interactions with other PD risk pathways and compensatory networks. Further studies in human and non-human primate systems will be required to define the molecular basis of these species-specific interactions and their contribution to PD pathogenesis.

## PINK1/parkin as potential therapeutic targets

The International Parkinson and Movement Disorder Society defines PD as a core motor syndrome characterized neuropathologically by SN neurodegeneration and synuclein deposition [157]. Knockdown of *PINK1* and *PRKN* in primate brain induces abnormal α-syn phosphorylation [123], a prodromal phenomenon, allowing exploration of downstream mechanisms.

Given that PINK1 and parkin have distinct expression and distribution patterns in the brain [117], it is important to separately investigate their primate-specific substrates. Parkin regulates proteins via ubiquitination and the proteasome system, such as regulation of microglial NLRP3 inflammasome activation [158]. PINK1 has broader functions, including substrates such as UBC12, Keap1, and Cullin3, linking it to neddylation and ER functions [110, 134].

These observations suggest that dysfunction of the PINK1/parkin axis affects multiple downstream pathways simultaneously. Consequently, restoring the pathway activity has emerged as an attractive therapeutic strategy. Clinical and experimental studies suggest that disruption of parkin Ser65 phosphorylation impairs parkin activation, leading to substrate accumulation and PD-related pathogenesis [159]. Increased insoluble parkin and Ser65-phosphorylated ubiquitin are observed in sporadic PD brains, suggesting dysregulation of parkin activation and homeostasis, supporting the therapeutic potential of enhancing parkin function [160, 161]. Consistently, recent biochemical studies have shown that parkin undergoes age-dependent shifts in solubility in the human brain, transitioning from soluble to detergent-insoluble forms after midlife [162], a process associated with oxidative modification of key cysteine residues and enhanced redox activity of parkin [163]. In parallel, parkin function is impaired in disease-associated mutants and during aging, contributing to increased cellular vulnerability to oxidative stress. Our recent research indicates that parkin S65 phosphorylation decreases with age in monkeys, accompanied by increased pS129-α-syn, suggesting that aging exacerbates parkin-related neurodegeneration [123]. Overexpression of wild-type parkin reduces α-syn accumulation, highlighting the therapeutic potential of targeting parkin phosphorylation [123, 164].

In MPTP-induced PD mouse model, parkin overexpression mitigates dopaminergic degeneration and improves outcomes [165]. In patient-derived neural progenitor cells, restoring parkin expression partially rescues transcriptomic and functional deficits [166]. Recent structural and pharmacological studies identified tetrahydroproparzolo-pyrazine compounds as molecular glues to stabilize parkin’s RING0 domain with phospho-ubiquitin, enhancing E3 ligase activity and partially rescuing the pathogenic Ubl domain-defective *parkin* mutants [167–169].

High-throughput screening identified FB231 as a putative parkin activator, but subsequent studies showed it acts as a mild mitochondrial stressor via iron chelation, sensitizing cells to mitophagy [170]. A similar mechanism is observed with deferiprone (DFP), which paradoxically accelerated disease progression in a Phase II PD trial, highlighting that chronic mitochondrial stress in humans may be toxic [171].

*PINK1* overexpression in aged monkey brains enhances parkin phosphorylation, reduces α-syn aggregation, and exerts neuroprotection [123]. *Pink1* overexpression also counteracts mutant huntingtin toxicity in *Drosophila* [172] and prevents tau hyperphosphorylation in a rat model of Alzheimer’s disease [149]. Small-molecule PINK1 activators, including Kinetin [173], KR ProTides [174], and MTK458 [175], have been identified. MTK458, a brain-penetrant activator, enhances mitophagy and clears α-syn aggregates in neurons and mouse models [175], and has completed Phase I trials (NCT06414798). While this paper [175] is currently available as a preprint, it provides important evidence relevant to the potential clinical development of PINK1-based therapeutics.

Despite these promising findings, recent studies have highlighted important challenges in the pharmacological activation of the PINK1/parkin pathway. Although compounds such as Kinetin, KR ProTides, and MTK458 have been described as PINK1 activators, accumulating evidence suggests that some of these agents may not function through direct kinase activation. Instead, they appear to engage the pathway by inducing mild mitochondrial stress, thereby stabilizing PINK1 and promoting downstream mitophagy. Recent evidence further suggests that MTK458 may also act as a weak mitochondrial toxin [170], indicating a potentially narrow therapeutic window. This distinction is therapeutically important because mitochondrial depolarization is both the physiological trigger of PINK1 activation and a potential source of neuronal injury. To provide a broader overview of these therapeutic strategies and their respective mechanistic challenges, representative compounds targeting different nodes of the PINK1/parkin pathway are summarized in Table 5, including direct/indirect activators, USP30 inhibitors, and mitochondrial modulators. The examples of FB231 and DFP illustrate this challenge, as chronic sensitization to mitochondrial stress may paradoxically exacerbate neurodegeneration despite enhancing mitophagy-related readouts. These findings suggest that increasing PINK1/parkin signaling is not necessarily equivalent to neuroprotection and highlight the need to distinguish true target engagement from stress-induced pathway activation.

**Table 5:** Summary of candidate therapeutic strategies targeting the PINK1/parkin pathway: mechanisms of action and safety challenges

Drug/molecule class	Representative compounds	Main mechanism of action	Activation mode	Development stage	Key safety concerns and translational challenges	References
Substrate mimetics	Kinetin, KR ProTides	Initially proposed as direct PINK1 activators; recent structural studies suggest that PINK1 may not directly utilize these compounds, and the precise mechanism remains to be clarified	Putative PINK1 activation (mechanism unresolved)	Preclinical	Multi-step metabolic activation required; modest potency; limited BBB penetration; insufficient in vivo efficacy data	[173, 176, 177]
Mitochondrial stress inducers	MTK458, FB231, Niclosamide, DFP (deferiprone)	Activate the PINK1/parkin pathway indirectly through mild mitochondrial stress, metabolic perturbation, or partial mitochondrial depolarization	Stress-dependent pathway activation	Phase I completed (MTK458); Preclinical (FB231); Approved drug repurposing (Niclosamide, DFP)	Indirect stress-dependent mechanism rather than true target engagement Narrow therapeutic window Potential cumulative mitochondrial toxicity with chronic treatment	[170, 171, 175, 177–180]
USP30 inhibitors	MTX325, FT3967385, CMPD-39, MF-094	Inhibit USP30-mediated deubiquitination, thereby enhancing PINK1/parkin-dependent mitochondrial ubiquitination and mitophagy	Pathway enhancer	Preclinical/early development	Limited brain penetration, potential compensatory mechanisms, and unknown long-term safety and clinical translatability	[181–185]
parkin activators	BIO-2007817	Promote formation of the open parkin conformation and enhance E3 ubiquitin ligase activity	Direct parkin activation	Preclinical	Selectivity toward parkin and potential off-target effects	[169]
Natural products/mitochondrial modulators	Salidroside, Celastrol	Multi-target regulation involving antioxidant responses, NRF2 signaling, and autophagy pathways	Indirect activation	Preclinical	Unclear primary targets and pleiotropic toxicity	[186–188]
Mitochondrial uncouplers or respiratory chain inhibitors	CCCP, FCCP, Valinomycin, Antimycin A, Oligomycin	Robust activation of the PINK1/parkin pathway through mitochondrial depolarization or inhibition of the respiratory chain	Stress-dependent pathway activation	Experimental research tools	Severe cytotoxicity; no realistic translational potential	[189–191]

Reference [175] is a preprint article

The profound phenotypic differences across species pose challenges for preclinical drug validation. Non-human primates, owing to their genetic and structural similarity to the human brain, provide a valuable platform for studying potential therapeutic targets within the PINK1–parkin axis. Future therapeutic avenues include: (1) gene therapy for PINK1/parkin overexpression; (2) small-molecule activators of the axis; (3) compounds targeting phosphorylated parkin; and (4) modulation of downstream substrates. Furthermore, excessive mitochondrial turnover in the absence of adequate mitochondrial biogenesis may ultimately compromise cellular bioenergetics, particularly in vulnerable dopaminergic neurons. Therefore, future therapeutic strategies should aim to restore mitochondrial quality control while preserving overall mitochondrial homeostasis. Reliable pharmacodynamic biomarkers will also be critical for clinical translation. Biomarkers such as phosphorylated ubiquitin (pS65-Ub), phosphorylated parkin (pS65-parkin), and other downstream pathway signatures may help assess on-target engagement, optimize dosing, and identify potential mitochondrial toxicity in human studies [192, 193].

Beyond the challenges of pharmacological validation, the development of robust preclinical models remains essential for assessing these therapeutic candidates. Given the high cost and long breeding time of non-human primates, human-derived brain organoids offer an alternative tool to investigate the PINK1/parkin axis and screen drugs, although they may not fully recapitulate age-dependent neuropathology. Brain organoids, three-dimensional self-organizing neural cultures derived from human pluripotent stem cells, recapitulate key aspects of human brain development, including cellular diversity, tissue architecture, and even early synaptic formation [194–196]. When generated from patients carrying *PINK1* or *PRKN* mutations, or from isogenic gene-edited lines, these organoids can model human-specific molecular pathways in a controlled culture environment. For instance, midbrain organoids enriched with dopaminergic neurons have been used to detect early mitochondrial deficits, impaired mitophagy, and α-syn accumulation in a human cellular context [197–200]. Moreover, organoids are amenable to high-content imaging and pharmacological screening, enabling rapid testing of compounds that restore PINK1–parkin function or bypass downstream deficits. However, current organoid models have notable limitations. Most cultures lack functional vasculature and immune components [201, 202], and they typically develop only to fetal-stage equivalents [201], limiting their ability to model late-onset or age-related neurodegenerative processes. Chronic stress, protein aggregation, and progressive neuronal loss seen in aged human brains remain difficult to reproduce. Nevertheless, when used in parallel with non-human primate models, brain organoids provide a powerful, scalable, and human-relevant platform for mechanistic dissection and early-stage drug discovery targeting the PINK1–parkin axis.

## Questions and future directions

**Fig. 4:**
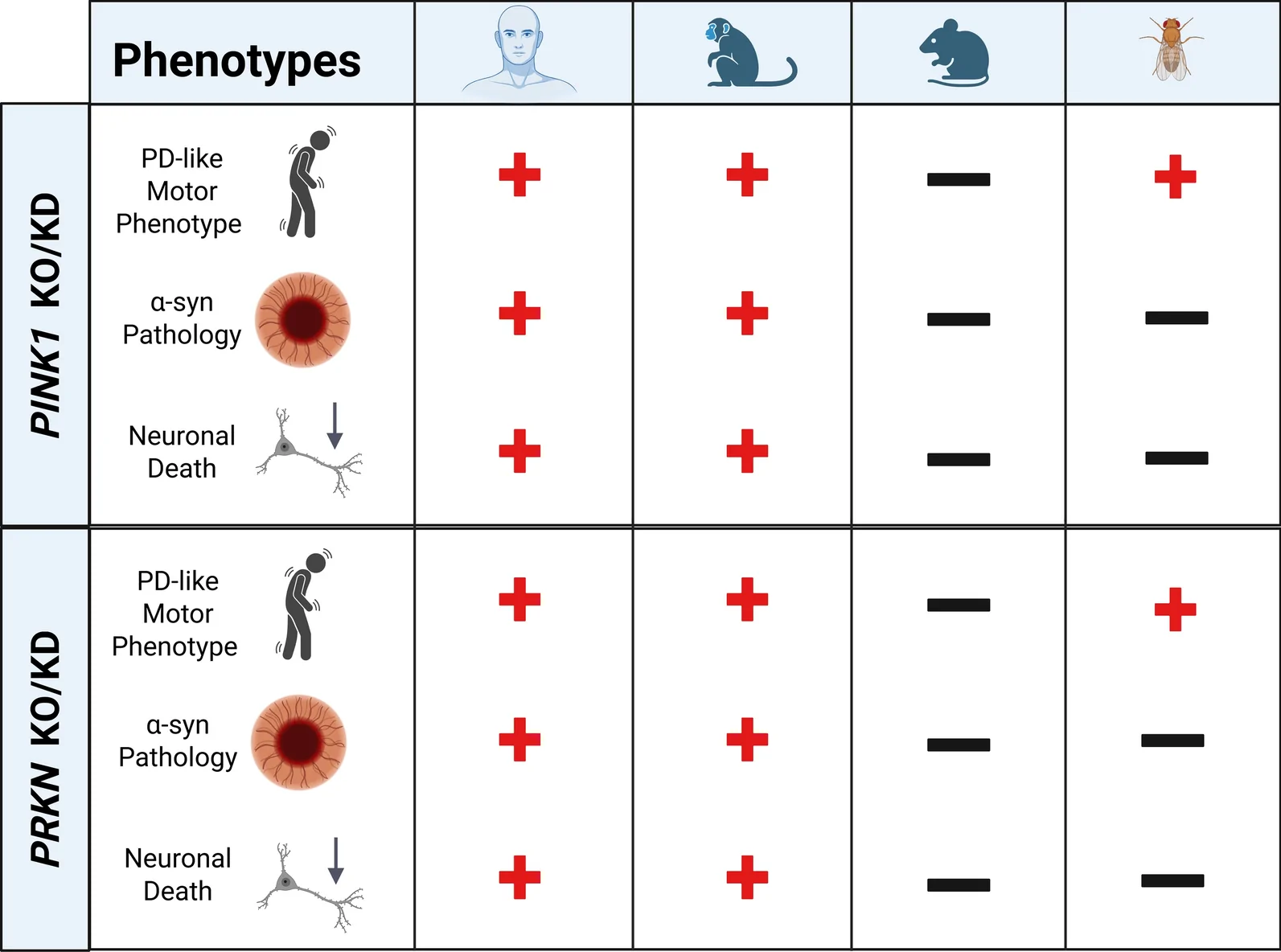
Species-specific phenotypic outcomes of PINK1 and parkin deficiency in *Drosophila*, mice, monkeys and humans. The table compares pathological phenotypes following *PINK1* or *PRKN* gene editing across species, where “+” and “−“ indicate the presence (+) or absence (−) of motor deficits, α-synuclein (α-syn) pathology, and neuronal death. This comparison highlights species-dependent differences in phenotypic outcomes and underscores their relevance to Parkinson's disease

Disrupting PINK1 or parkin in different animal models results in pronounced phenotypic differences (Fig. 4). Fly studies established that PINK1 acts upstream of parkin [36, 70, 71]. In rodents, basal PINK1 expression is extremely low, and single-gene knockouts do not cause severe mitochondrial abnormalities or dopaminergic neuron loss [54, 76, 93]. In non-human primates, however, loss of PINK1 or parkin leads to significant neurodegeneration and motor dysfunction, resembling human PD [120–123]. This cross-species difference may stem from selective PINK1 expression in primate brains [122]. Non-human primate studies also demonstrate that PINK1 phosphorylates parkin in vivo and that defective phosphorylation leads to α-syn accumulation, a PD hallmark [123]. These findings suggest that targeting PINK1–parkin phosphorylation may offer new neuroprotective strategies against α-syn pathology. In addition to the biological insights gained from non-human primate models, several technical and conceptual limitations should also be acknowledged.

First, technical considerations associated with genome editing should be acknowledged when interpreting findings from non-human primate models. In embryonic genome-editing approaches, CRISPR/Cas9-mediated mosaicism may arise during early development, resulting in heterogeneous mutations across tissues and cell populations [203–205]. Although AAV-mediated somatic gene manipulation in the adult brain avoids developmental compensation that may occur in germline models, this spatially and temporally controlled manipulation is limited by restriction to specific brain regions. Moreover, brain regional phenotypic interpretation may be influenced by factors such as regional variability in viral transduction, heterogeneous editing efficiency among targeted neurons, and differences in the extent of gene disruption across individual animals. Off-target effects remain a longstanding concern associated with gene editing-mediated knockout strategies. While current studies employing whole-genome sequencing, deep sequencing, and VIVO-based analyses have reported limited evidence of substantial off-target editing [206], comprehensive evaluation of editing specificity remains an important consideration for future studies. Continued improvements in guide RNA design [207] and high-fidelity Cas9 platforms [208] are likely to further enhance the accuracy and the reliability of genome engineering in primates [209].

Second, while non-human primate models provide unique opportunities to investigate PD in a system that more closely resembles the human brain, several challenges remain. Ethical considerations, long reproductive cycles, and high maintenance costs inevitably restrict cohort sizes, which may limit statistical power and behavioral analysis. Furthermore, although existing models reproduce important neuropathological and motor phenotypes, PD is now recognized as a multisystem disorder with a long prodromal phase and non-motor symptoms [210]. Reproducing non-motor symptoms, autonomic and gastrointestinal dysfunction, peripheral pathology, and disease progression in animal models over extended periods therefore remains an important goal for future studies. Integrating longitudinal phenotyping with molecular and biomarker-based analyses may help further enhance the translational relevance of these models.

Third, beyond scientific and technical considerations, ethical and regulatory challenges in utilizing non-human primates warrant continued attention. Although non-human primate models provide unparalleled opportunities to investigate disease mechanisms in a system closely related to humans, their use raises important ethical concerns regarding animal welfare, experimental justification, and long-term oversight. Addressing these issues through transparent ethical review, rigorous experimental design, and responsible governance will be essential for ensuring the sustainable and socially acceptable advancement of non-human primate-based PD research.

Despite recent advances, several fundamental questions regarding the physiological functions of the PINK1/parkin axis in primate brains remain unresolved. First, how is PINK1 more stably expressed in primate brains compared to mice and pigs? Since *PINK1* mRNA is uniformly present across species, regulatory mechanisms must govern PINK1 protein processing or stability in primates. Second, how does the PINK1/parkin axis protect against neurodegeneration in primate brains? The absence of abnormal mitochondrial accumulation in PINK1-deficient monkeys [122] and in postmortem PD brains with PINK1/parkin mutations [211] implies that the PINK1–parkin axis confers neuroprotection via mitochondria-independent pathways. Third, how does the PINK1/parkin axis participate in clearing toxic α-syn? Finally, what are the independent functions of PINK1 and parkin beyond their axis activity?

Addressing these questions will require experimental systems that faithfully capture the unique biology of the primate brain. Because non-human primates uniquely express endogenous PINK1 under physiological conditions, they represent an essential model for investigating these unresolved issues. Human brain organoids offer a complementary, scalable platform for modeling patient-specific genetic backgrounds and screening potential therapeutics, although they cannot fully recapitulate the complex physiological environment of the primate brain.

Accordingly, rather than replacing existing experimental systems, non-human primate models should be viewed as a critical component of a broader translational research pipeline. The integration of human organoids, genetically engineered rodent models, and non-human primate models is likely to provide the most comprehensive strategy for bridging molecular mechanisms with clinical phenotypes while balancing scientific, ethical, and practical considerations. Ultimately, resolving these questions will be crucial for understanding the physiological functions of the PINK1/parkin axis in the brain and for developing therapeutic strategies targeting PINK1/parkin dysfunction in PD.

## Data Availability

No datasets were generated or analysed during the current study.

## Abbreviations

AAV9: Adeno-associated virus serotype 9
α-syn: α-synuclein
DFP: Deferiprone
EOPD: Early-onset Parkinson’s disease
ER: Endoplasmic reticulum
KO: Knockout
MDVs: Mitochondrial-derived vesicles
PD: Parkinson’s disease
SN: Substantia nigra
TH: Tyrosine hydroxylase
Ubl: Ubiquitin-like domain
